# Supplementary figures and images for: Homocysteine thiolactone contributes to the prognostic value of fibrin clot structure/function in coronary artery disease
Source: PLoS One. 2022 Oct 27;17(10):e0275956. doi: 10.1371/journal.pone.0275956 (PMC9612472; doi:10.1371/journal.pone.0275956)

## Slide 1
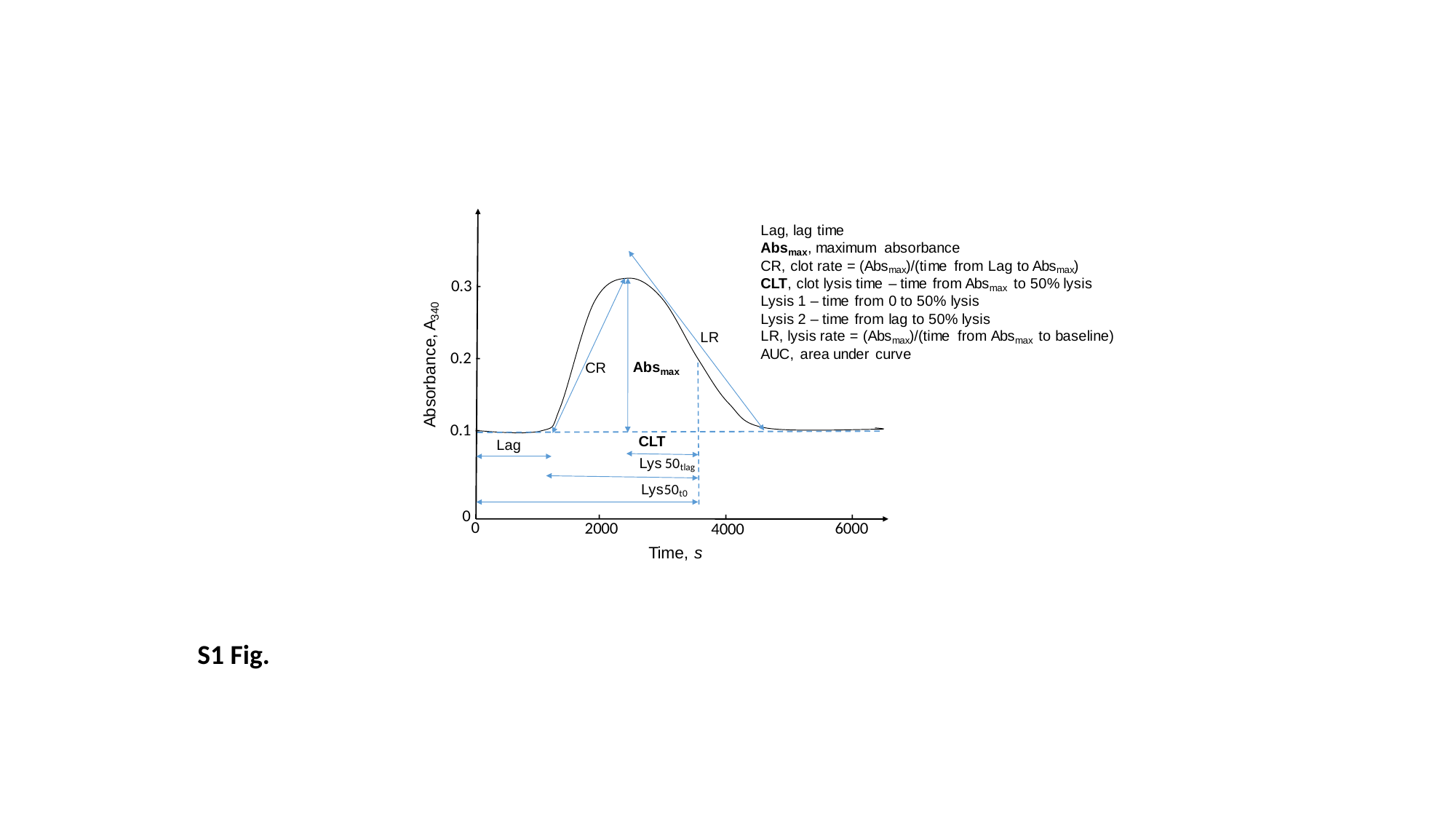

S1 Fig.

Supplement: S1 Fig — Variables examined in a greater detail in the present study, CLT and Absmax, are highlighted in bold. (PPTX) [file pone.0275956.s001.pptx]

## Slide 1
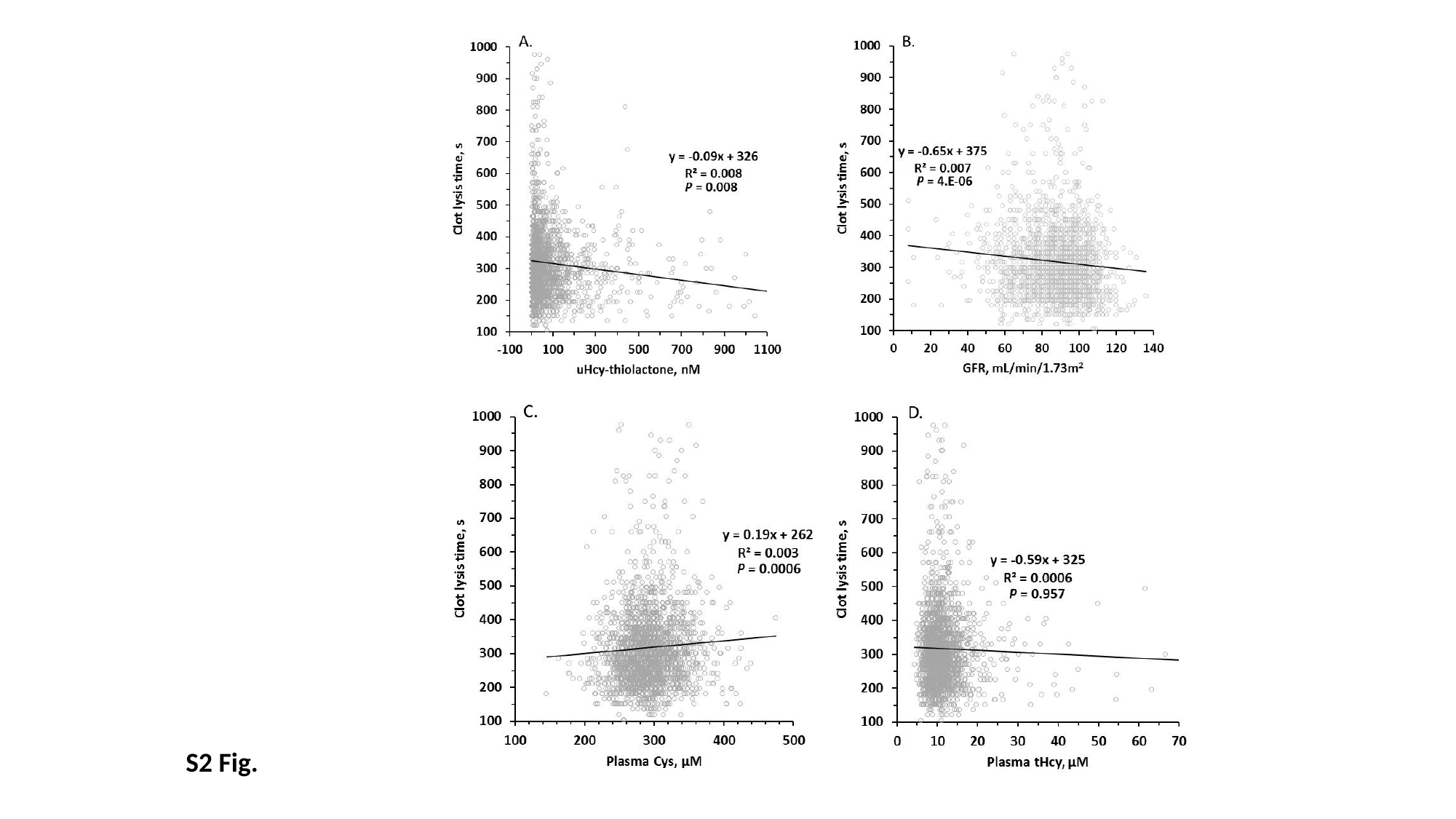

S2 Fig.

Supplement: S2 Fig — Relationships between CLT and plasma Cys (A.), tHcy (B.), uHcy-thiolactone (C.) and GFR (D.). Spearman P values are shown. uHcy-thiolactone was present in each sample and varied from 1.3 to 1,724.0 nM. (PPTX) [file pone.0275956.s002.pptx]

## Slide 1
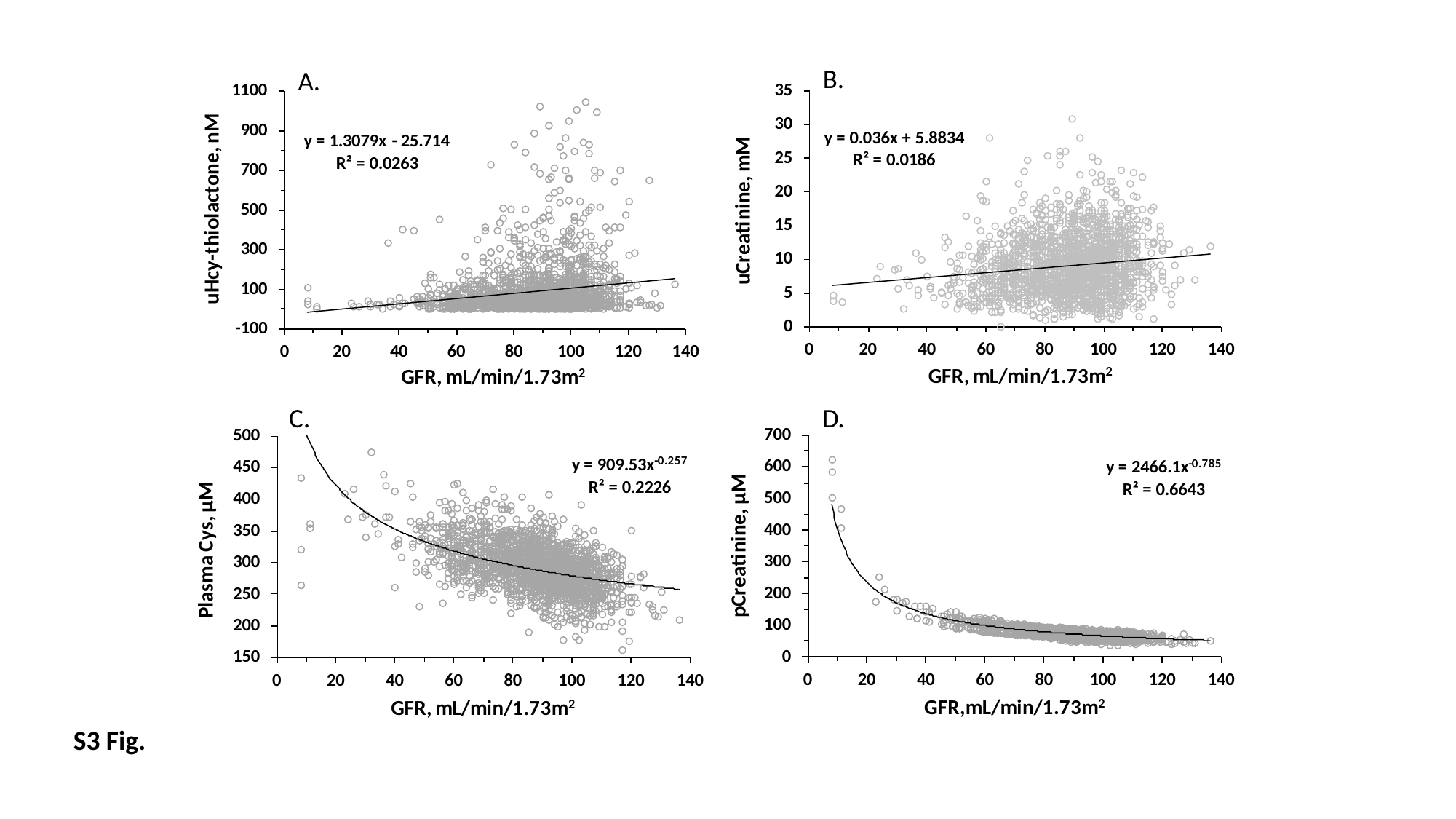

S3 Fig.

Supplement: S3 Fig — Relationships between GFR and uHcy-thiolactone (A.), uCreatinine (B.), plasma Cys (C.) and pCreatinine (D.). (PPTX) [file pone.0275956.s003.pptx]
