# Supplementary material for: Homocysteine thiolactone contributes to the prognostic value of fibrin clot structure/function in coronary artery disease
Source: PLoS One. 2022 Oct 27;17(10):e0275956. doi: 10.1371/journal.pone.0275956 (PMC9612472; doi:10.1371/journal.pone.0275956)
Supplement: S1 Table — Values are from analyses of data for n = 1,983 patients. Nomenclature is after Carter et al. [2]. P<0.000, except when indicated otherwise. The clotting and lysis variables are illustrated in S1 Fig. *Terms Absmax and CLT referring to terms MaxAbs and Lysis50MA, respectively, of Carter et al. [2], have been use in the present study. (DOCX) [file pone.0275956.s004.docx]

**S1 Table.** **Pearson correlation coefficients for relationships between turbidimetric clotting and lysis variables in the WENBIT cohort of CAD patients.** Values are from analyses of data for n = 1,983 patients. Nomenclature is after Carter *et al*., *Arterioscler Thromb Vasc Biol* 2007; 27:2783-2789.  *P*<0.000, except when indicated otherwise. The clotting and lysis variables are illustrated in **S1** **Fig**. *Terms **Abs_max_** and **CLT** referring to terms MaxAbs and Lysis50_MA_, respectively, of Carter *el al*. ATVB 2007, have been use in the present study.

|  | MaxAbs, **Abs_max_*** | Clot Rate | Lys50_t0_ | Lys50_tlag_ | Lysis50_MA_, **CLT*** | Lysis Rate | LysisArea  AUC |
| --- | --- | --- | --- | --- | --- | --- | --- |
| Lag | **-0.61** | **-0.54** | **0.46** | **-0.20** | **-0.08** (*P*=0.001) | **0.41** | **0.05** (*P*=0.028) |
| AbsMax,  **Abs_max_*** |  | **0.87** | **-0.17** | **0.26** | **0.23** | **-0.69** | **0.27** |
| Clot Rate |  |  | **-0.41** | **-0.07** (*P*=0.002) | 0.00 (*P*=0.899) | **-0.67** | **0.16** |
| Lys50_t0_ |  |  |  | **0.77** | **0.73** | **0.28** | **0.24** |
| Lys50_tlag_ |  |  |  |  | **0.86** | 0.02  (*P*=0.407) | **0.23** |
| Lysis50_MA_, **CLT**^b^ |  |  |  |  |  | **0.05** (*P*=0.028) | **0.29** |
| Lysis Rate |  |  |  |  |  |  | **-0.11** |
