# Supplementary material for: Homocysteine thiolactone contributes to the prognostic value of fibrin clot structure/function in coronary artery disease
Source: PLoS One. 2022 Oct 27;17(10):e0275956. doi: 10.1371/journal.pone.0275956 (PMC9612472; doi:10.1371/journal.pone.0275956)
Supplement: S2 Table — (DOCX) [file pone.0275956.s005.docx]

**S2 Table. Descriptive statistics of the variables analyzed in the present study.**

| Variable | Men (*n*=1,396) | | Women (*n*=556) | | *P* value |
| --- | --- | --- | --- | --- | --- |
|  | Mean | SD | Mean | SD |  |
| uHcy-thiolactone, nM | 96.8 | 141.6 | 69.1 | 104.5 | 0.000 |
| Cys, μM | 291.5 | 35.3 | 295.7 | 41.2 | 0.024 |
| Vitamin E, mg/L | 30.9 | 7.3 | 32.3 | 7.6 | 0.201 |
| BMI, kg/m^2^ | 26.3 | 3.8 | 26.4 | 5.0 | 0.887 |
| GFR, mL/min/1.73 m^2^ | 88.9 | 16.2 | 84.2 | 16.5 | 0.000 |
| uCreatinine, mM | 9.8 | 4.3 | 7.1 | 3.6 | 0.000 |
| Abs_max_ , A_340_ | 0.088 | 0.042 | 0.084 | 0.043 | 0.049 |
| Fibrinogen, g/L | 3.64 | 3.70 | 0.72 | 0.70 | 0.073 |
| Albumin, g/L | 43.3 | 42.7 | 2.4 | 2.5 | 0.000 |
| Triglycerides, mM | 1.82 | 1.61 | 1.38 | 0.98 | 0.001 |
| tHcy, μM | 11.9 | 5.8 | 10.8 | 4.0 | 0.000 |
| pCreatinine, μM | 81.4 | 28.9 | 67.6 | 22.7 | 0.000 |
| CRP, mg/L | 3.62 | 7.80 | 3.96 | 6.05 | 0.336 |
| LDL-C, mM | 3.13 | 1.01 | 3.19 | 1.01 | 0.235 |
| HDL-C, mM | 1.23 | 0.32 | 1.48 | 0.41 | 0.000 |
| ApoA1, mg/dL | 1.29 | 0.23 | 1.48 | 0.27 | 0.000 |
| Lpa, mg/dL | 0.40 | 0.38 | 0.46 | 0.43 | 0.004 |
| ApoB, mg/dL | 0.91 | 0.25 | 0.93 | 0.25 | 0.204 |
| Age, years | 61.2 | 10.4 | 63.2 | 10.4 | 0.000 |
| CLT, s | 313.3 | 131.6 | 328.9 | 137.6 | 0.019 |
