# Supplementary material for: Homocysteine thiolactone contributes to the prognostic value of fibrin clot structure/function in coronary artery disease
Source: PLoS One. 2022 Oct 27;17(10):e0275956. doi: 10.1371/journal.pone.0275956 (PMC9612472; doi:10.1371/journal.pone.0275956)
Supplement: S3 Table — *Groups of patients without and with AMI or mortality events are indicated by 0 and 1, respectively. (DOCX) [file pone.0275956.s006.docx]

**S3 Table. Groups of CAD patients stratified by outcome and cutoff values of clot lysis time (CLT) or maximum absorbance (Abs_max_).** * Groups of patients without and with AMI or mortality events are indicated by 0 and 1, respectively.

| Outcome group |  | Clot lysis time (CLT) | | | Maximum absorbance (Abs_max_) | | |
| --- | --- | --- | --- | --- | --- | --- | --- |
|  | No. of patients (%) | CLT cutoff value | No. of patients ≤cutoff (%) | No. of patients >cutoff (%) | CLT cutoff value | No. of patients ≤cutoff (%) | No. of patients >cutoff (%) |
|  | | | | | | | |
| AMI* | 2006 | 397.5 | 1637 | 345 | 0.025 | 131 | 1852 |
| 0 | 1846 (92.0) |  | 1516 (92.6) | 306 (88.7) |  | 127 (96.9) | 1696 (91.6) |
| 1 | 160 (8.0) |  | 121 (**7.4**) | 39 (**11.3**) |  | 4 (**3.1**) | 156 (**8.4**) |
|  | | | | | | | |
| Mortality* | 2006 | 532.5 | 1890 | 92 | 0.169 | 1916 | 67 |
| 0 | 1890 (94.2) |  | 1786 (94.5) | 80 (87.0) |  | 1808 (94.4) | 59 (88.1) |
| 1 | 116 (5.8) |  | 104 (**5.5**) | 12 (**13.0**) |  | 108 (**5.6**) | 8 (**11.9**) |
